# High-Temperature Annealing of Random Telegraph Noise in a Stacked CMOS Image Sensor After Hot-Carrier Stress †

**DOI:** 10.3390/s26010282

**Published:** 2026-01-02

**Authors:** Calvin Yi-Ping Chao, Thomas Meng-Hsiu Wu, Charles Chih-Min Liu, Shang-Fu Yeh, Chih-Lin Lee, Honyih Tu, Zhong-Da Wu, Joey Chiao-Yi Huang, Chin-Hao Chang

**Affiliations:** Taiwan Semiconductor Manufacturing Company (TSMC), Hsinchu 30077, Taiwan; mhwuzd@tsmc.com (T.M.-H.W.); cmliut@tsmc.com (C.C.-M.L.); sfyehe@tsmc.com (S.-F.Y.); clleeza@tsmc.com (C.-L.L.); hytu@tsmc.com (H.T.); zdwua@tsmc.com (Z.-D.W.); joey_huang@tsmc.com (J.C.-Y.H.); cchangr@tsmc.com (C.-H.C.)

**Keywords:** CMOS image sensor (CIS), random telegraph noise (RTN), hot-carrier stress (HCS), hot-carrier injection (HCI), MOSFET device aging and reliability

## Abstract

This paper studies the temperature effects on device aging, particularly the random telegraph noise (RTN) degradation and the threshold voltage ($V_{t}$) shift in a stacked CMOS image sensor (CIS) caused by hot-carrier stress (HCS). Measurements indicate that both are worse when stressed at lower temperatures. Further, the RTN traps generated by HCS can be deactivated effectively by a subsequent high-temperature annealing at 240 °C for up to 360 min. In contrast, the RTN traps in chips not stressed by hot carriers are essentially unaffected by annealing at the same temperature for the same amount of time. This suggests that the physical structure of the RTN traps caused by process-induced damage (PID) without HCS might be different from that generated by HCS. The exact microscopic nature of the differences between these two kinds of RTN traps is not clear and requires further investigation. This work also suggests that RTN degradation could be a useful indicator for device aging for reliability testing and modeling.

## 1. Introduction

Device aging and reliability are important issues for all semiconductor products. CMOS image sensors (CISs) are no exception [1]. Some well-known aging mechanisms include hot-carrier injection (HCI), bias temperature instability (BTI), time-dependent dielectric breakdown (TDDB), and electron migration (EM) [2,3,4,5,6,7,8,9]. In this work, we focus on HCI-induced device degradation and recovery.

As the pixel pitch of state-of-the-art CISs continues to shrink down to the range of 0.4 to 0.5 microns [10,11,12,13,14], it is inevitable that the number of photons captured by a pixel reduces accordingly. Therefore, it becomes increasingly challenging to maintain a high full-well capacity and a low readout noise to meet the targets of dynamic range and signal-to-noise ratio. Random telegraph noise (RTN) is one of the major components of the low-frequency noises in CISs. However, statistical studies of RTN, especially in the context of device aging, are seldom reported in the literature.

Previously, we reported the increase in RTN caused by hot-carrier stress (HCS) in a stacked CIS at room temperature [15,16]. We pointed out the notable differences between the RTN degradation and the transistor $V_{t}$ shift commonly monitored in reliability testing. In this study, the HCS temperatures are extended to cover a wider range from −35 °C to 120 °C. Furthermore, we investigate the effects of high-temperature annealing on RTN after HCS. By analyzing the time-domain noise waveforms, all pixels in an 8.3 Mpixel array are sorted into two categories, RTN and non-RTN. Then, we trace the changes in the noise behavior of each pixel throughout a series of stress and annealing experiments. The results lead to an important observation that the RTN traps generated by HCS can be annealed effectively by high-temperature treatment, but the RTN traps caused by PID cannot be eliminated easily.

Despite the fact that RTN in MOSFET has been known for several decades, there is no consensus on the origins of RTN traps. The microscopic structures of RTN traps are still up for debate. The mechanisms of the generation and annihilation of RTN traps are not fully understood. The existence of different categories of RTN traps that some can be systematically eliminated by high-temperature annealing and some cannot is neither predicted nor explained by existing atomic models or ab initio theoretical calculations. The goal of this paper is to provide experimental evidence to emphasize this observation. This is supported by a statistical analysis of 1M devices. To our knowledge, the key points we highlighted have not been reported or answered before. We hope this work will stimulate further studies on the nature of RTN traps.

## 2. Materials and Methods

### 2.1. Test Chip Design and Characteristics

The test chip is a two-layer stacked and backside-illuminated (BSI) CMOS image sensor with an 8.3 Mpixel (2512^V^ × 3296^H^) resolution and a 0.8 µm pixel pitch. The top layer consists of a pixel array, fabricated in a 1P4M 45 nm CIS process. The bottom layer consists of analog readout circuits and a digital processor, fabricated in a 1P7M 22 nm mixed-mode process. The two layers are face-to-face stacked by a wafer-level hybrid bond (HB) technology. The array has a 4^V^ × 2^H^ shared-pixel structure and is read out by 1648 12-bit column ADCs with front amplifiers supporting 1 to 8 analog gains. The average DNL of the ADCs is within ±0.5 DN (digital number). The average INL is less than 0.5% of the full signal range.

**Figure 1:**
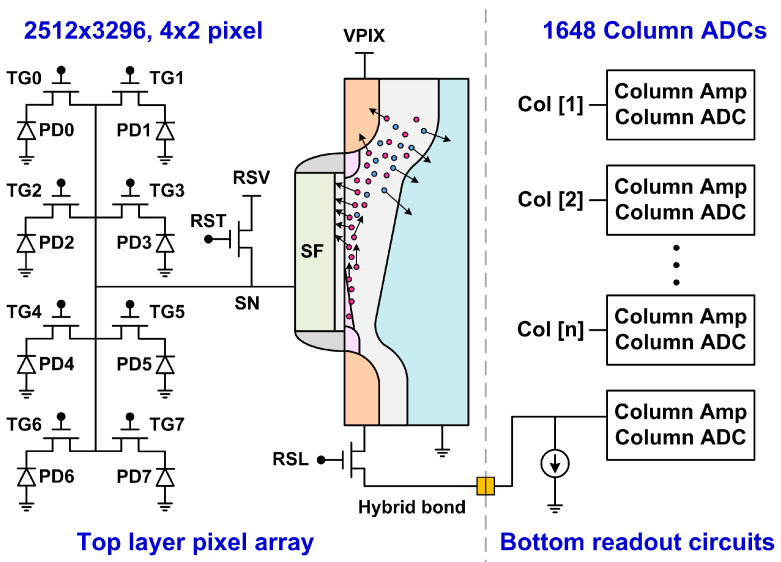
A simplified test chip signal chain and an illustration of the hot-carrier stress condition, where the red circles represent electrons and the blue circles represent holes. The device under stress is the SF. PD0–7 indicate the photodiodes, and TG0–7 indicate the transfer gates in each pixel group. The total number of SF is 628 × 1648 = 1.03 M.

A simplified chip architecture is shown in Figure 1. The three pixel devices (RST, RSL, SF) can be operated up to 3.3 V. The device under test (DUT) is the source follower (SF) NMOS with W = 0.16 µm, L = 0.87 µm, and a 5.7 nm dielectric thickness, biased by a constant current source of 7.2 µA in normal operations. The conversion factor from the ADC output to the SF input is 292 µV/DN at gain of 1 and 36.5 µV/DN at gain of 8. The median read noise (including an amplifier at a gain of 1 and ADC) is about 190 µV-rms operated at a 60 MHz clock and a 1.48 fps frame rate. The random noise (RN) is measured at gain of 8. The threshold voltage shift (${∆V}_{t}$) is measured at gain of 1. Both are calculated back to input-referred values at the SF gates. Throughout this paper, the CIS chip is operated in the dark under a test mode where the transfer gates (TGs) are disabled, and the SF input is fixed by an external voltage via the RSV terminal such that photon shot noises and dark leakage are not involved. The measured outputs are primarily SF temporal noises.

### 2.2. Hot-Carrier Injection and the Temperature Dependence

**Figure 2:**
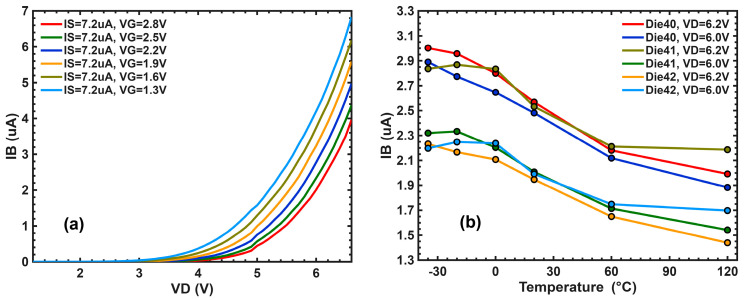
The substrate current $I_{B}$ is a key indicator of hot-carrier injection. (**a**) $I_{B}$ as a function of $V_{D}$ stepped by $V_{G}$ at room temperature, where $I_{S}$ is biased at 7.2 µA. (**b**) The temperature dependence of $I_{B}$ at $V_{D}=$ 6.0 and 6.2 V, where $V_{G}$ is fixed at 2.8 V.

Under normal imaging operations, the SF drain voltage ($V_{D}≝V_{PIX}$) is set to 3.1 V. When $V_{PIX}$ is increased, the channel conduction electrons are accelerated by the lateral electric fields and gain kinetic energies. The energetic (hot) electrons can, in turn, generate more electron–hole pairs through impact ionization, manifested as a rapid increase in the substrate current ($I_{B}$) due to unrecombined holes. When the electron energy exceeds the electron affinity difference between Si and SiO_2_ (about 3.25 eV), it can jump over or tunnel through the Si-SiO_2_ barrier to damage the oxide. In this experiment, the source voltage ($V_{S}$) of the SF is about 1.5 V. A significant increase in substrate current is observed when $V_{PIX}$ exceeds 5.5 V, i.e., when the $V_{DS}$ of the SF is higher than 4 V. Figure 2 shows the data measured from the SF devices in separate test keys.

### 2.3. Hot-Carrier Stress and High-Temperature Annealing Experiments

In hot-carrier stress experiments, $V_{PIX}$ is raised to 6 V, and the temperatures are maintained at −35 °C, 0 °C, 20 °C, 60 °C, and 120 °C for each selected sample. The time intervals for the series of stresses are 10, 20, 50, 100, 200, and 400 min. In this paper, the accumulated stress times (i.e., 0, 10, 30, 80, 180, 380, and 780 min) are used as the reference time. Before, after, and between two consecutive stresses, the samples are measured at $V_{PIX}=3.1$ V at room temperature. In annealing experiments, the stressed samples are annealed in normal laboratory ambient air at temperatures of 120 °C, 160 °C, 200 °C, and 240 °C for a sequence of accumulated annealing times of 10, 30, 60, 160, and 360 min under power-off conditions. Similarly, the samples are measured at room temperature before, after, and between two consecutive annealing processes under normal operating conditions. The ΔRN value caused by the stress and annealing is calculated as $\Delta RN≝\sqrt{{RN\left(t_{s}\;\mathrm{or}\;t_{a}\right)}^{2}-{RN\left(t_{s}=0\right)}^{2}}$, where $t_{s}$ and $t_{a}$ denote the accumulated stress and annealing time, respectively.

We have verified that the RTN of a stressed sample does not show any noticeable relaxation effects at room temperature up to a few weeks.

## 3. Results

### 3.1. Temperature Dependence of the Hot-Carrier Stress

**Figure 3:**
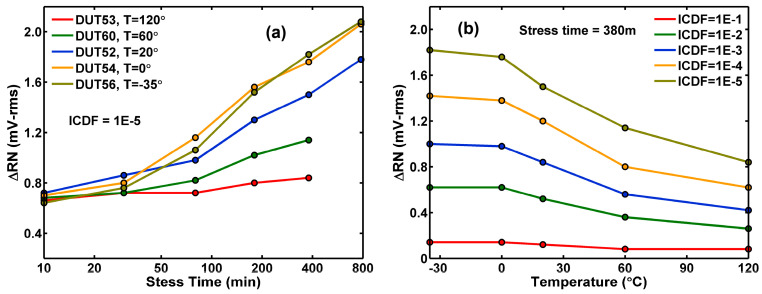
(**a**) ΔRN value at the distribution tail (ICDF = 10^−5^) as a function of stress time from −35 °C to 120 °C. (**b**) Constant-ICDF contours of ΔRN after a stress of 380 min versus temperature.

The ΔRN value at the 10 ppm tail of the inverse cumulative distribution function (ICDF) versus stress time for temperatures ranging from −35 °C to 120 °C is plotted in Figure 3a. It shows that the degradation is worse at lower temperatures. Figure 3b shows that the temperature dependence of various constant-ICDF contours is similar and consistent.

The RN degradation and the $V_{t}$ shift are correlated to the amount of injected hot electrons and proportional to the measured substrate currents, which show a similar temperature dependence in Figure 2 and Figure 3. This behavior is consistent with what has been reported in the literature [17,18,19].

However, the trend is not universal. Later studies [20,21] indicate that the HCS degradation is higher at lower temperatures only for long-channel devices (approximately L > 100 nm). The trend is opposite for short-channel devices (L < 100 nm) due to different mechanisms. The SF under test has L = 870 nm; therefore, it is a long-channel device. For the rest of this article, we selected the device stressed at −35 °C for recovery experiment. For high-temperature annealing, we selected the results of 240 °C for discussion.

### 3.2. Effects of Stress and Annealing on Random Noise Distributions

**Figure 4:**
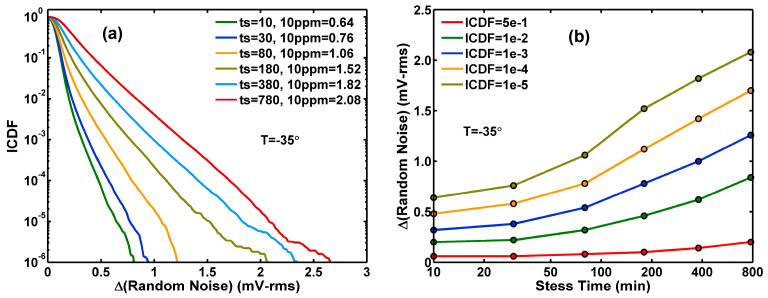
Hot-carrier stress at −35 °C. (**a**) A family of ICDF curves of ΔRN for stress times ranging from 10 to 780 min. (**b**) A family of ΔRN constant-ICDF contours as functions of the stress time.

**Figure 5:**
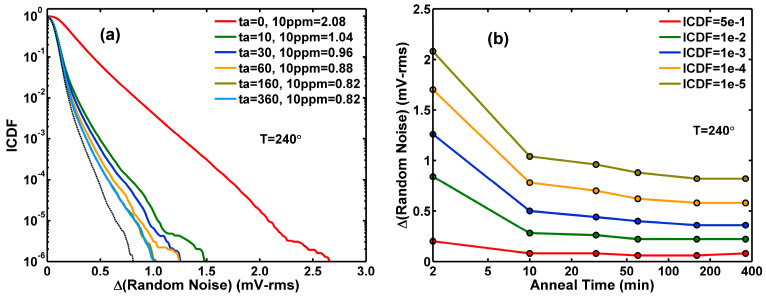
High-temperature annealing at 240 °C. (**a**) A family of ICDF curves of ΔRN for annealing times ranging from 10 to 360 min. The black dotted curve corresponds to the beginning of the stress. (**b**) A family of ΔRN constant-ICDF contours as functions of the annealing time.

Figure 4a shows the gradual change in the ΔRN ICDF curves for a stress time ($t_{s}$) ranging from 10 to 780 min. The ΔRN values at 10 ppm levels are shown in the legends and plotted in Figure 4b along with several different ICDF values versus stress times. After the HCI stress, the same sample is annealed at 240 °C for up to 360 min. Figure 5a,b shows the ΔRN change over the annealing time ($t_{a}$). Note that all measurements are performed at room temperature between two consecutive stress and annealing processes. Comparing Figure 5 to Figure 4, we can see that annealing is almost like a reversed process of stress. This means that the damage delivered to the device by HCI stress can be mostly repaired by annealing. The speed of the recovery is relatively faster during the first 10 min at 240 °C. However, the recovery is close to but not 100% complete. We can see this by comparing the ICDF curve at the end of the annealing ($t_{a}=$ 360) to the curve at the beginning of the stress ($t_{s}=$ 10), which is shown as the black dotted curve in Figure 5a.

**Figure 6:**
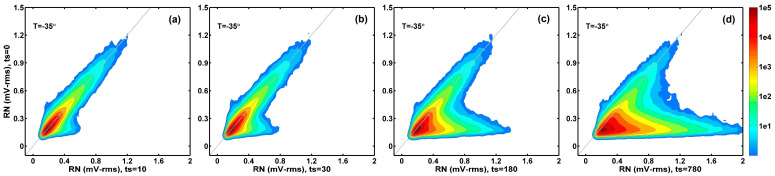
Correlation between the RN value after stress and the RN value before stress. A total of (**a**) 10 min of stress; (**b**) 30 min of stress; (**c**) 180 min of stress; and (**d**) 780 min of stress.

**Figure 7:**
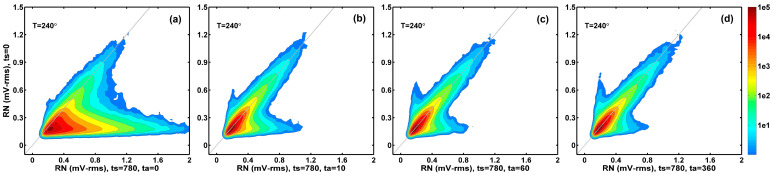
Correlation between the RN value after annealing and the RN value before stress. A total of (**a**) 0 min of annealing; (**b**) 10 min of annealing; (**c**) 60 min of annealing; and (**d**) 360 min of annealing.

The details in the ΔRN changes during the stress and annealing experiments can be seen clearly in 2D correlation histograms between RN at each stage of stress (or annealing) compared to the initial condition. Figure 6 compares the RN value at $t_{s}=$ 10, 30, 180, and 780 min to the RN value before stress ($t_{s}=$ 0). Figure 7 compares the RN value at $t_{a}=$ 10, 10, 60, and 360 min after 780 min of stress to the RN value before stress ($t_{s}=$ 0). As pointed out previously [15,16], the prominent feature in the 2D histograms is that there are two distinct groups of devices. The devices centering around the x = y diagonal line (gray color) show small RN changes. On the other hand, the group of devices in the lower-right branch of the 2D histogram contribute to a large portion of the RN changes. They are mostly the RTN devices generated by the stress and recovered in the subsequent annealing, which is discussed in the next paragraph. The annealing process in Figure 7 is like a time reversal of the degradation process in Figure 6.

**Figure A1:**
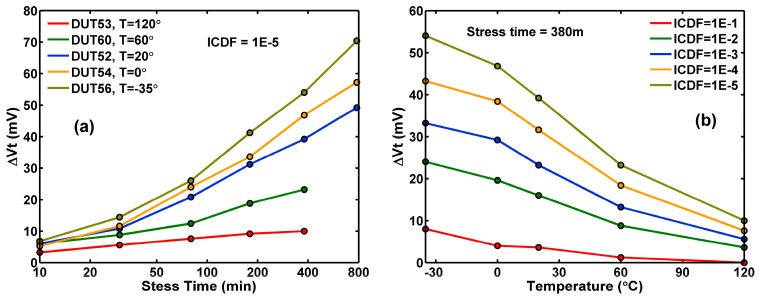
(**a**) $\Delta V_{t}$ at the distribution tail (ICDF = 10^−5^) as a function of stress time from −35 °C to 120 °C. (**b**) Constant-ICDF contours of $\Delta V_{t}$ after 380 min of stress as a function of temperature.

**Figure A2:**
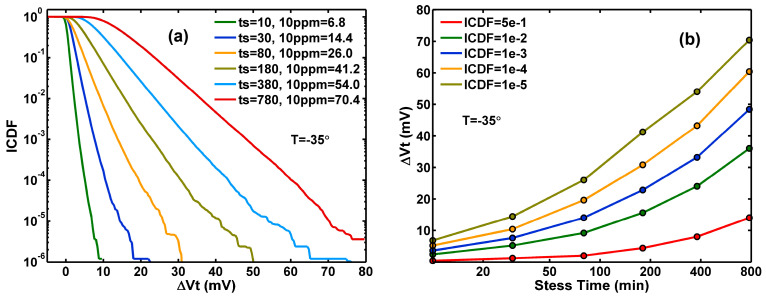
Hot-carrier stress at −35 °C. (**a**) A family of ICDF curves of $\Delta V_{t}$ for stress times ranging from 10 to 780 min. (**b**) A family of $\Delta V_{t}$ constant-ICDF contours as functions of the stress time.

**Figure A3:**
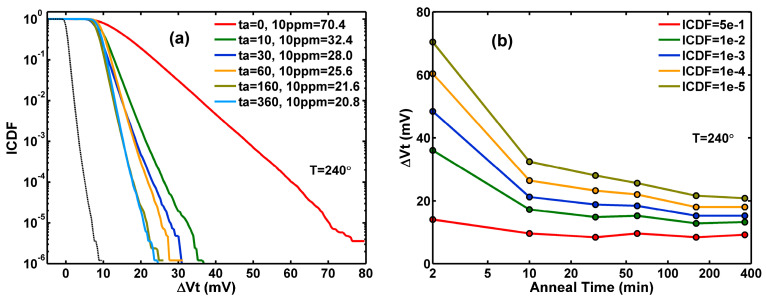
High-temperature annealing at 240 °C. (**a**) A family of ICDF curves of $\Delta V_{t}$ for annealing times ranging from 10 to 360 min. The black dotted curve corresponds to the beginning of the stress. (**b**) A family of $\Delta V_{t}$ constant-ICDF contours as functions of the annealing time.

**Figure A4:**
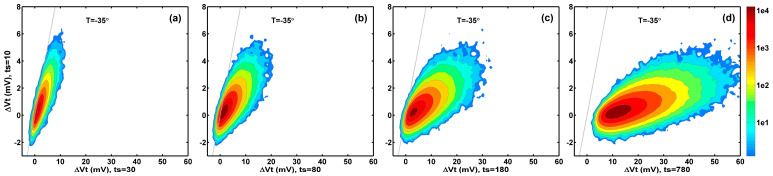
Correlation between $\Delta V_{t}$ after stress and $\Delta V_{t}$ before stress. A total of (**a**) 10 min of stress; (**b**) 30 min of stress; (**c**) 180 min of stress; and (**d**) 780 min of stress.

**Figure A5:**
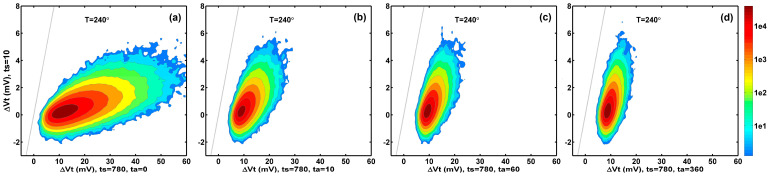
Correlation between $\Delta V_{t}$ after annealing and $\Delta V_{t}$ before stress. A total of (**a**) 0 min of annealing; (**b**) 10 min of annealing; (**c**) 60 min of annealing; and (**d**) 360 min of annealing.

In conventional device aging and reliability testing, the threshold voltage shift (${∆V}_{t}$) plays a more prominent role than RN degradation. In this study, both are characterized at the same time and compared side by side. Their physical origins are closely related, and some of the aging behaviors are similar. Although ${∆V}_{t}$ is not at the center of the following discussion, we include the results in Appendix A as a complementary reference. There is a one-to-one correspondence between Figure A1, Figure A2, Figure A3, Figure A4 and Figure A5 and Figure 3, Figure 4, Figure 5, Figure 6 and Figure 7, respectively.

### 3.3. Classification of the Random Noise Types

For CIS process and product development purposes, RN histograms and ICDF plots are sufficient to evaluate the RTN performance from wafer to wafer and from lot to lot. A common practice [22,23,24] is to set an empirical RN threshold and calculate the percentage of pixels above the threshold as an RTN performance index. Apparently, this is a simplification for the convinience of benchmarking. In real silicon, we find that the distribution of RTN pixels in the full array is more complicated.

In this paper, we follow the methodology developed in previous work [15,16,25,26] to classify the pixel RN types according to their time-domain noise waveforms. In general, the known pixel noises in the dark are the thermal noises, the flicker noises, and the RTN, assuming the reset KTC noises are suppressed by a true CDS (correlated double sampling) operation. The pixels showing multiple identifiable discrete levels in noise waveforms are classified RTN pixels. The pixels with nearly ideal Gaussian noise distributions are considered non-RTN pixels. There is a gray area between these two types of noises. Some pixels do not show mutliple discrete levels but the noise distributions significantly deviate from the Gaussian forms. We tentatively call these RTN-like pixels (e.g., Figure 12 of [16]).

**Figure 8:**
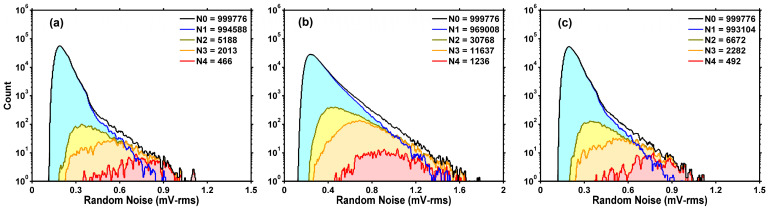
Sorting results of all devices in three stages: (**a**) before stress, (**b**) after stress, and (**c**) after annealing. The meaning of the numbers $N_{0},\;N_{1},\;N_{2},\;N_{3}$, and $N_{4}$ are defined in the text.

Figure 8a–c show the result of sorting all the pixels before stress, after stress, and after annealing, respectively. In the legend, $N_{4}$ is the number of pixels showing three or more discrete levels; $N_{3}$ is the number of pixels showing two or more discrete levels; $N_{2}$ equals $N_{3}$ plus the number of RTN-like pixels; $N_{1}$ is the number of non-RTN pixels; and $N_{0}$ is the total number of pixels. Since eight pixels share one SF, which dominates the readout noise, the total number of SFs in Figure 8 is about one-eighth of 8.3 million. In the rest of this paper, we treat $N_{2}$ as the number of generalized RTN pixels. In other words, each pixel is classified as either RTN or non-RTN (i.e., $N_{0}=N_{1}+N_{2}$). From Figure 8, we can see that it is not possible to choose a well-defined threshold to separate RTN pixels from non-RTN pixels.

### 3.4. Key Findings

The separation of RTN and non-RTN devices allows us to keep track of the change in behavior of the individual devices throughout the stress and anneal experiments. In Table 1, the numbers of RTN and non-RTN devices before stress, after stress, and after annealing are summarized. There are eight possible experimental outcomes. Each outcome can be labeled by a 3-bit binary code as the case number, where a “1” means RTN and a “0” means non-RTN. For instance, one device in case 010 means that it is non-RTN before stress, turns into RTN after stress, and changes back to non-RTN after annealing.

**Figure 9:**
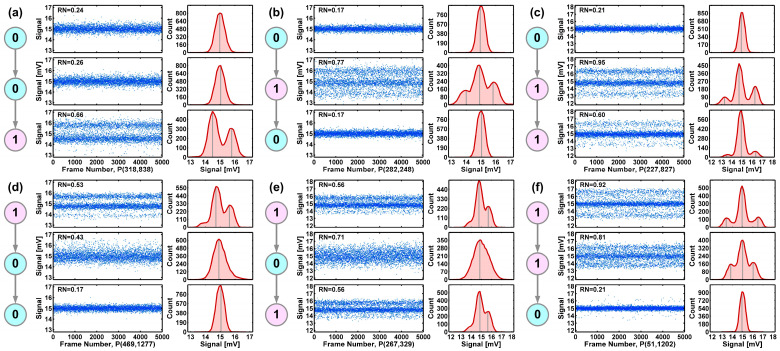
Six selected examples corresponding to (**a**) case 001, (**b**) case 010, (**c**) case 011, (**d**) case 100, (**e**) case 110, and (**f**) case 110. The noise waveforms and histograms before stress, after stress, and after annealing are shown in top, middle, and bottom panels of each figure, respectively.

The first observation is that the cause-and-effect is not as simple as one might expect. Intuitively, we might anticipate that the effect of HCS is to generate RTN traps. However, the result shows that HCS could not only turn some non-RTN devices into RTN devices but could also turn some RTN devices into non-RTN devices. Vice versa, the annealing also works both ways. The underlying physical process seems complicated. It indicates that the results are not entirely deterministic but depend on certain probabilities. To visualize and to confirm the transitions, six examples are selected and plotted in Figure 9, excluding the trivial no-change cases 000 and 111.

Secondly, although there are devices in all the eight possible cases, the most outstanding one is obviously case 010 with the most transitions. In the initial condition before stress, there are 5188 RTN devices, or 0.5% of the total population. After 780 min of stress at −35 °C, 28,388 devices are turned from non-RTN to RTN, about 5.5 times the initial RTN number. Among these 28,388 RTN devices, 27,856 return to being non-RTN after annealing at 240 °C for 360 min. The recovery rate is about 98%. This indicates that the RTN traps generated by HCS can be effectively deactivated by high-temperature annealing.

**Figure 10:**
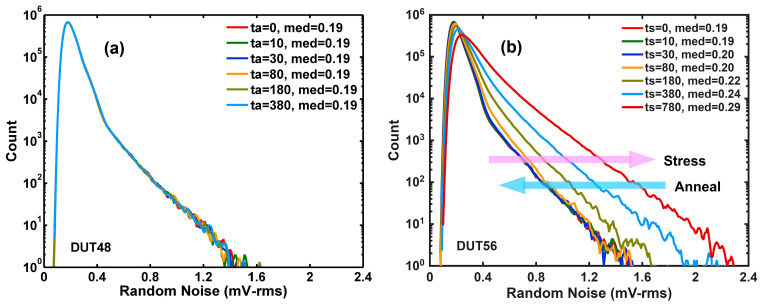
(**a**) DUT48 (unstressed) has a comparable performance to DUT56 before stress. The RN distributions are not changed by high-temperature annealing. (**b**) DUT56 (stressed) shows a degraded RN histogram that is restored to its initial condition after annealing, indicating deactivation of HCS-generated RTN traps. In the legends, “med” denotes the median of RN.

Thirdly, two distinct types of RTN traps are observed. “Soft” RTN traps, generated by HCS, are readily annealed at 240 °C, while “hard” RTN traps, caused by PID, are resistant to annealing. The contrast is evident by comparing the RN histograms of an unstressed sample to those of a stressed sample. Figure 10a shows that the RN histograms of an unstressed sample remain unchanged after 240 °C annealing for up to 360 min, indicating that PID-induced RTN traps are largely unaffected. Figure 10b shows that for a stressed sample, HCS significantly degrades the RN histograms due to new RTN traps. Most of the new RTN traps are deactivated by high-temperature annealing and the initial RN distribution is nearly restored.

## 4. Discussion

RTN traps are, in general, related to defects introduced into the gate dielectric or bulk Si during a long sequence of manufacturing steps. For instance, some could be due to high-energy ion implantation, and some could be related to reactive ion etching. It is often difficult to pinpoint the exact source and eliminate RTN traps completely. In the CIS chips we characterized, the percentage of RTN pixels in a large array is small, typically in the order of 0.1% to 0.5%. PID-induced RTN traps are stable in room temperature for several months or even years. In this study, we find that they cannot be easily deactivated by high-temperature annealing. One explanation is that at the time of fab-out, the RTN traps have survived the high-temperature treatment in the backend-of-line (BEOL) process up to a few hundreds of degrees Celsius for hours; therefore, they cannot be annealed further. They may be tentatively called “hard” RTN traps.

On the other hand, HCS generates many new and relatively “soft” RTN traps, which can be easily eliminated by a subsequent 240 °C annealing. This important distinction among different RTN traps has not been pointed out in the literature to the best of our knowledge.

Several studies have linked HCI-induced interface states and oxide traps to the dissociation of Si-H bonds, which creates Si dangling bonds [21,27,28,29,30,31,32,33,34]. Reversely, the annealing of these traps at high temperature could be explained by hydrogen re-passivating the Si dangling bonds. Hydrogen and hydrogen-related chemicals are commonly used in many semiconductor fabrication processes. Once introduced, the hydrogen atom can diffuse easily through the Si and oxide lattice. The nominal Si-H bond strength is about 3.6 eV; however, some reports have shown that the Si-H bond can be broken by hot electrons of energies much less than 3.6 eV [33,34].

The defects in bulk SiO_2_ and on the Si-SiO_2_ interface have many complicated forms such as Si dangling bonds ($P_{b}$ centers), oxygen vacancies (*E*’ centers), hydroxyl-*E*’ centers, hydrogen bridges, peroxyl radicals, and non-bridging oxygen hole centers [35,36,37]. Electronic excitation or charge trapping on some of these sites may alter their atomic configurations and electrical characteristics. It is possible that the differences between hard and soft RTN traps are related to the atomic structural differences in the traps, although our macroscopic data cannot lead to any specific microscopic pictures.

## 5. Conclusions

We point out one key difference between native or built-in RTN traps in samples unstressed after fab-out and RTN traps created by hot-carrier stress. The latter can be mostly eliminated by 240 °C annealing in ambient air for a few hours, while the former cannot be annealed easily. The underlying physics is not clarified yet. We hope that this study can stimulate further investigations, perhaps by atomic structure calculation and simulation, to better understand the dynamics and mechanisms of the generation and annihilation of RTN traps.

## Figures and Tables

**Table 1:** The number of RTN and non-RTN devices before stress, after stress, and after annealing. The eight possible outcomes are labeled by case numbers of 3 binary bits, where “1” represents RTN and “0” represents non-RTN. RTN data are labelled by pink background color and non-RTN data are labelled by light blue color.

Before Stress		After Stress		After Annealing		Case
RTN	5188	RTN	2380	RTN	2314	111
				Non-RTN	66	110
		Non-RTN	2808	RTN	2047	101
				Non-RTN	761	100
Non-RTN	994,588	RTN	28,388	RTN	532	011
				Non-RTN	27,856	010
		Non-RTN	966,200	RTN	1779	001
				Non-RTN	964,421	000

## Data Availability

Data is contained within the article.

## Abbreviations

The following abbreviations are used in this manuscript:

CIS	CMOS Image Sensor
BSI	Backside-Illuminated
RN	Random Noise
RTN	Random Telegraph Noise
HCS	Hot-Carrier Stress
HCI	Hot-Carrier Injection

## Appendix A

In conventional reliability testing and modeling, the threshold voltage shift ($∆V_{t}$) is one of the most important parameters. On the other hand, RN degradation (∆RN) is not routinely monitored. Nevertheless, in real devices, these two aging effects always happen hand in hand. The physical causes of both effects are related to the generation of oxide traps and interface states. In this work, $∆V_{t}$ and ∆RN are characterized at the same time during the stress and annealing process. In the main text, we focus on the discussion of ∆RN. For completeness, the $∆V_{t}$ data are presented here in Figure A1, Figure A2, Figure A3, Figure A4 and Figure A5 as counterparts of Figure 3, Figure 4, Figure 5, Figure 6 and Figure 7 on ∆RN. From the standpoint of statistical distributions and the temperature dependencies, the $∆V_{t}$ values in Figure A1, Figure A2 and Figure A3 are very similar to the ∆RN values in Figure 3, Figure 4 and Figure 5. However, the evolution of the correlation plots in Figure A4 and Figure A5 are notably different from Figure 6 and Figure 7, as previously pointed out [14,15]. Unlike Figure 6 and Figure 7, the 2D histograms in Figure A4 and Figure A5 do not show two distinct branches. The degradation and recovery of $∆V_{t}$ happen more uniformly to the entire population, while the ∆RN value is significantly larger for RTN devices than that for non-RTN devices.
